# Combination therapy of midodrine and droxidopa for refractory hypotension in heart failure with preserved ejection fraction per a pharmacist’s proposal: a case report

**DOI:** 10.1186/s40780-021-00193-z

**Published:** 2021-03-03

**Authors:** Yuki Asai, Tomoaki Sato, Daisuke Kito, Takanori Yamamoto, Iwao Hioki, Yasuhisa Urata, Yasuharu Abe

**Affiliations:** 1 Pharmacy, National Hospital Organization Mie Chuo Medical Center, 2158-5 Hisaimyojincho, Tsu, Mie 514-1101 Japan; 2 Department of Cardiovascular Surgery, National Hospital Organization Mie Chuo Medical Center, 2158-5 Hisaimyojincho, Tsu, Mie 514-1101 Japan

**Keywords:** Chronic heart failure, Combination therapy, Droxidopa, Heart failure with preserved ejection fraction, Heart failure with reduced ejection fraction, Hypotension, Midodrine

## Abstract

**Background:**

Patients with chronic heart failure (CHF) are often treated using many diuretics for symptom relief; however, diuretic use may have to continue despite hypotension development in these patients. Here, we present a case of heart failure with preserved ejection fraction (HFpEF), which is defined as ejection fraction ≥50% in CHF, and refractory hypotension, which was treated with midodrine and droxidopa to normalize blood pressure.

**Case presentation:**

The patient was a 62-year-old man with a history of HFpEF due to mitral regurgitation and complaints of dyspnea on exertion. He had been prescribed multiple medications at an outpatient clinic for CHF management, including azosemide 60 mg/day, bisoprolol 2.5 mg/day, enalapril 2.5 mg/day, spironolactone 50 mg/day, and tolvaptan 15 mg/day. The systolic blood pressure (SBP) of the patient remained at 70–80 mmHg because the use of the diuretic could not be reduced or discontinued owing to edema and weight gain. He was hospitalized for the exacerbation of CHF. Although midodrine 8 mg/day was administered to improve hypotension, the SBP of the patient increased only up to 90 mmHg. On the 35th day after hospitalization, the urine volume decreased significantly (< 100 mL/day) due to hypotension. When droxidopa 200 mg/day replaced intravenous noradrenaline on the 47th day, the SBP remained at 100–120 mmHg and the urine volume increased.

**Conclusions:**

Oral combination treatment with midodrine and droxidopa might contribute to the maintenance of blood pressure and diuretic activity in HFpEF patients with refractory hypotension. However, further long-term studies evaluating the safety and efficacy of this combination therapy for patients with HFpEF are needed.

## Background

While it is well-known that diuretic treatment is crucial to improve the prognosis and symptoms among patients with chronic heart failure (CHF) [1–3], a diminished diuretic response is common in these patients, increasing the required diuretic dose [4]. Hypotension has been defined as systolic blood pressure (SBP) < 90 mmHg and/or diastolic blood pressure (DBP) < 60 mmHg [5]. In particular, diuretic-induced hypotension causes dizziness [6]. However, the administration of diuretics in these patients cannot be stopped as this would likely result in the progression of heart failure [7].

Many reports have shown that droxidopa, a noradrenaline (NA) prodrug, improves the symptoms of orthostatic hypotension in patients with Parkinson disease, multiple system atrophy, and pure autonomic failure [8–10]. While extensive evidence is available regarding neurogenic hypotension, information on the efficacy and safety of droxidopa for refractory hypotension with CHF is insufficient. Midodrine is widely used for the management of orthostatic blood pressure [11]; however, there is no evidence available on the efficacy of the combination of midodrine and droxidopa. In recent years, drug treatment has been reported to improve prognosis in heart failure with reduced ejection fraction (HFrEF), in which ejection fraction (EF) is < 40% [1–3]. However, guidelines for the treatment of heart failure with preserved ejection fraction (HFpEF), in which EF is ≥50%, are not available. Here, we describe the case of a patient with HFpEF who was successfully treated for refractory hypotension.

## Case presentation

**Fig. 1:**
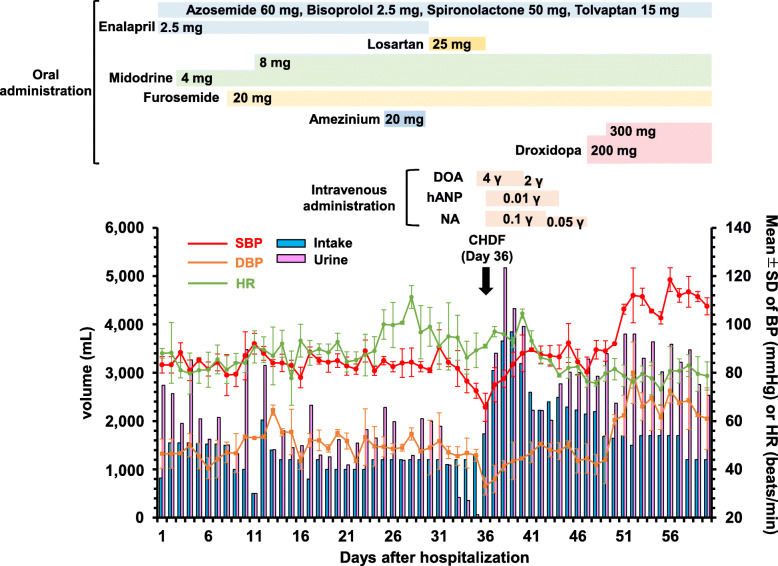
Clinical course of the hospitalized patient in this study. Up-titration and down-titration of medications while in the hospital are shown, along with the daily urine volume, intake volume, blood pressure, and HR. BP, blood pressure; CHDF, continuous hemodiafiltration; DBP, diastolic blood pressure; DOA, dopamine; hANP, human atrial natriuretic peptide; HR, heart rate; NA, noradrenaline; SBP, systolic blood pressure. The unit *γ* shows μg/kg/min. SBP, DBP, and HR are shown as the mean ± standard deviation (SD)

The patient was a 62-year-old man with a history of CHF due to mitral regurgitation and complaints of dyspnea on exertion (New York Heart Association functional class III). After mitral annuloplasty, he was prescribed multiple medications at an outpatient clinic for the management of CHF, including azosemide 60 mg/day, bisoprolol 2.5 mg/day, enalapril 2.5 mg/day, spironolactone 50 mg/day, and tolvaptan 15 mg/day. The SBP of the patient remained at 70–80 mmHg because diuretic use could not be reduced or discontinued due to the possible effects of edema and weight gain. He was hospitalized for exacerbation of heart failure. On admission, his SBP and DBP were 83 and 47, respectively, and his heart rate (HR) was 88 beats/min. On the 3rd day after hospitalization, a pharmacist proposed midodrine 4 mg/day, an oral pressor with a weak effect on HR [12], to the attending doctor, after which drug administration was started (Fig. 1). The EF was measured on the 8th day and was 53.4%, which is categorized as HFpEF. Furosemide 20 mg/day was started because the urine volume was low on the 8th day. Over a 9-day period after the initiation of midodrine treatment, the dose was increased to 8 mg/day; however, SBP increased only up to 90 mmHg. Although amezinium 20 mg/day was administered on the 25th day for further pressor action, it was discontinued on the 29th day due to the onset of tachycardia (Fig. 1).

**Fig. 2:**
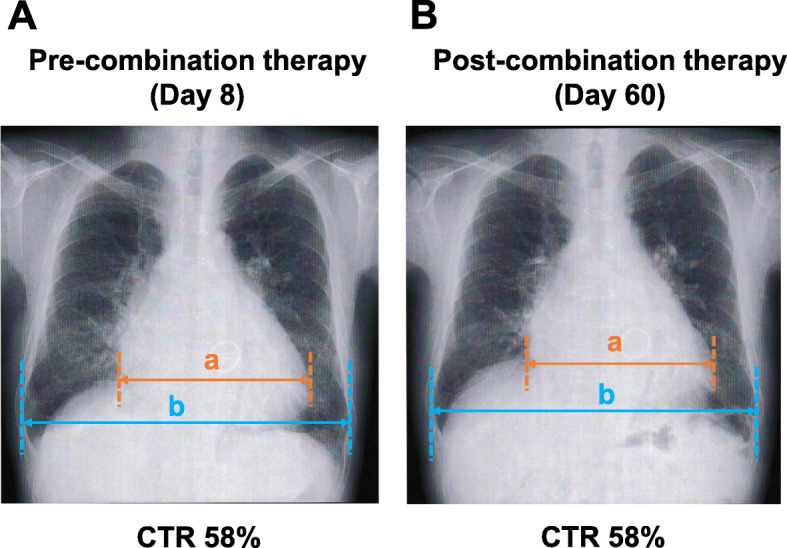
Chest X-ray of the patient pre- and post-combination therapy with midodrine and droxidopa. Cardiothoracic ratio (CTR, %) was calculated as (a/b) × 100. **A** Chest X-ray showing pre-combination therapy status on the 8th day of hospitalization with a CTR of 58%. **B** Chest X-ray showing post-combination therapy status on the 60th day of hospitalization with a CTR of 58%

In HFrEF, enalapril has been shown to contribute to improved prognosis [13], whereas it is unknown if this effect is present in HFpEF. However, because in HFpEF it may also be highly beneficial to continue with renin-angiotensin system inhibitors, we changed the drug regimen to losartan, which is reported to have a weak hypertensive effect among the angiotensin II receptor blockers [14]. From the 35th day of hospitalization, blood pressure decreased and urine volume decreased significantly (< 100 mL/day), and losartan was discontinued on the 36th day. Consequently, the patient underwent continuous hemodiafiltration (CHDF) on the 36th day only. As shown in Fig. 1, continuous intravenous infusion of dopamine from the 35th day and NA and human atrial natriuretic peptide from the 36th day gradually increased the blood pressure and urine volume. However, it was suggested that it would not be possible to maintain blood pressure upon NA discontinuation. Therefore, the attending doctor consulted a pharmacist regarding the switch from NA to oral pressor drugs. On the basis of some case reports [8–10], the pharmacist suggested switching from intravenous NA to droxidopa, which is converted to NA in vivo, on the 47th day. When the dosage of droxidopa was increased from 200 mg/day to 300 mg/day on the 49th day of hospitalization, SBP and DBP increased to 100–120 mmHg and 60–80 mmHg, respectively. As blood pressure increased, urine volume could be maintained at an average of 3000 mL/day. Seven days after the start of this combination therapy, the EF was 60.1% (Day 53), and no decrease was observed compared to the findings on the Day 8 (EF = 53.4%). In addition, this combination therapy also did not affect cardiothoracic ratio (CTR) (Day 8: CTR = 58% and Day 60: CTR = 58%) (Fig. 2). After discharge, the patient’s SBP and DBP were maintained using a combination of midodrine 8 mg/day and droxidopa 300 mg/day therapy, and his dizziness disappeared.

## Discussion and conclusions

Hypotension is one of the most serious side effects of diuretics in patients with CHF [6]. It causes not only dizziness, but also reduction of diuretic activity because of decreased blood flow [4]. Therefore, it is suggested that improving hypotension may contribute to ensuring diuretic responsiveness. In the case of our patient with HFpEF and refractory hypotension, combination therapy of midodrine and droxidopa increased blood pressure and improved diuretic responsiveness.

While a *β*-blocker may have potential to improve prognosis in HFpEF [15], bisoprolol decreases blood pressure [16]. In this case, because bisoprolol was used to control tachycardia, we continued to administer bisoprolol at the lowest possible dose while monitoring the HR.

Midodrine is an oral alpha-1 adrenergic agonist that acts as a blood pressor [11] and decreases HR [12]. Although the sample size was small, it was also reported that midodrine elevates EF significantly in HFrEF [17]. Considering this evidence, midodrine might be considered suitable as an oral pressor for patients with HFpEF. Midodrine can elevate SBP and DBP by approximately 20 mmHg [17], and the degree of increase in blood pressure in our patient was similar to that reported previously [17]. Although amezinium was administered on the 25th day, the HR of the patient increased (Fig. 1). Katoh et al. [18] revealed that amezinium elevated HR. Tachycardia is known to be an exacerbating prognostic factor for heart failure [19], suggesting that midodrine, but not amezinium, may show efficacy as an oral pressor in patients with HFpEF.

Despite the administration of midodrine, the urine volume decreased due to excessive hypotension on the 34th and 35th days of hospitalization, and CHDF was performed on the 36th day. It was speculated that the blood pressure might be insufficiently maintained. Because droxidopa is metabolized by L-aromatic-amino-acid decarboxylase to NA, which mediates a pressor response [20], it may be useful to switch from intravenous to oral NA treatment due to hypotension. As expected, up-titration of droxidopa from 200 to 300 mg/day combined with 8 mg/day midodrine rapidly and significantly improved hypotension. Because the blood pressure of the patient could be maintained using this combination therapy, it is considered that the responsiveness to diuretics increased. Therefore, it may be possible to reduce the dose of diuretics such as furosemide. In a double-blind, 4-period crossover study, there were no clinically relevant effects of droxidopa on HR [21]. While the cardiovascular safety of droxidopa has been reported in patients with neurogenic hypotension [22], the detailed safety profile in patients with a history of HFpEF remains unknown. No adverse effects of the combination therapy were noted over the short term in this case. To the best of our knowledge, there is no information regarding the efficacy and safety of droxidopa combined with midodrine in HFpEF patients over the long term. Accordingly, further studies evaluating the safety and efficacy of long-term combination therapy of droxidopa and midodrine for HFpEF patients are needed.

Based on our findings, the combination therapy of midodrine and droxidopa might be safely and effectively administered to HFpEF patients with refractory hypotension, but further studies need to be conducted. In general, diuretic use should be reduced or discontinued if hypotension develops in patients with CHF. If the administration of diuretics must be continued owing to CHF progression, it is advisable to first start midodrine and then add droxidopa if hypotension cannot be effectively controlled.

## Data Availability

All the data generated or analyzed in this study are included in the published article.

## Abbreviations

CHDF: Continuous hemodiafiltration
CHF: Chronic heart failure
CTR: Cardiothoracic ratio
DBP: Diastolic blood pressure
EF: Ejection fraction
HFpEF: Heart failure with preserved ejection fraction
HFrEF: Heart failure with reduced ejection fraction
HR: Heart rate
NA: Noradrenaline
SBP: Systolic blood pressure
